# Laninamivir Octanoate and Artificial Surfactant Combination Therapy Significantly Increases Survival of Mice Infected with Lethal Influenza H1N1 Virus

**DOI:** 10.1371/journal.pone.0042419

**Published:** 2012-08-01

**Authors:** Masaya Fukushi, Makoto Yamashita, Tohru Miyoshi-Akiyama, Shuku Kubo, Kenji Yamamoto, Koichiro Kudo

**Affiliations:** 1 Disease Control and Prevention Center, National Center for Global Health and Medicine, Tokyo, Japan; 2 Deputy Director-General's Laboratory, Research Institute, National Center for Global Health and Medicine, Tokyo, Japan; 3 Department of Virology, Institute of Biomedical and Health Sciences, Hiroshima University, Hiroshima, Japan; 4 Biological Research Laboratories, Daiichi Sankyo Co., Ltd., Tokyo, Japan; 5 Department of Infectious Diseases, Research Institute, National Center for Global Health and Medicine, Tokyo, Japan; The University of Hong Kong, China

## Abstract

**Background:**

Patients with influenza virus infection can develop severe pneumonia and acute respiratory distress syndrome (ARDS) which have a high mortality. Influenza virus infection is treated worldwide mainly by neuraminidase inhibitors (NAIs). However, monotherapy with NAIs is insufficient for severe pneumonia secondary to influenza virus infection. We previously demonstrated that mice infected with a lethal dose of influenza virus develop diffuse alveolar damage (DAD) with alveolar collapse similar to that seen in ARDS in humans. Additionally, pulmonary surfactant proteins were gradually increased in mouse serum, suggesting a decrease in pulmonary surfactant in the lung. Therefore, the present study examined whether combination therapy of NAI with exogenous artificial surfactant affects mortality of influenza virus-infected mice.

**Methodology/Principal Findings:**

BALB/c mice were inoculated with several viral doses of influenza A/Puerto Rico/8/34 (PR8) virus (H1N1). The mice were additionally administered exogenous artificial surfactant in the presence or absence of a new NAI, laninamivir octanoate. Mouse survival, body weight and general condition were observed for up to 20 days after inoculation. Viral titer and cytokine/chemokine levels in the lungs, lung weight, pathological analysis, and blood O_2_ and CO_2_ pressures were evaluated. Infected mice treated with combination therapy of laninamivir octanoate with artificial surfactant showed a significantly higher survival rate compared with those that received laninamivir octanoate monotherapy (*p* = 0.003). However, virus titer, lung weight and cytokine/chemokine responses were not different between the groups. Histopathological examination, a hydrostatic lung test and blood gas analysis showed positive results in the combination therapy group.

**Conclusions/Significance:**

Combination therapy of laninamivir octanoate with artificial surfactant reduces lethality in mice infected with influenza virus, and eventually suppresses DAD formation and preserves lung function. This combination could be effective for prevention of severe pneumonia secondary to influenza virus infection in humans, which is not improved by NAI monotherapy.

## Introduction

Influenza viruses, including highly pathogenic avian influenza (H5N1) and A(H1N1)pdm09 viruses, can induce viral pneumonia in humans [1]–[7]. In some cases, pneumonia becomes severe and develops into acute respiratory distress syndrome (ARDS), which is a fatal human respiratory disorder defined by four criteria [8]–[10]. In the lungs of patients with ARDS, diffuse alveolar damage (DAD) is frequently observed, which is characterized histopathologically by hyaline membranes lining the alveoli and alveolar ducts [11]–[13]. The factors that are responsible for development of ARDS/DAD from viral pneumonia remain uncertain. In addition, an effective treatment for severe respiratory failure has not yet been established [14]–[16]. Several neuraminidase inhibitors (NAIs) are available and have a therapeutic effect if used within 48 h of the appearance of symptoms. However, their effect is thought to be limited for severe pneumonia and ARDS/DAD secondary to influenza virus infection [17], [18]. Therefore, additional treatments in combination with NAIs have been attempted; e.g., extracorporeal membrane oxygenation [19], polymyxin B-immobilized fiber column [18], [20], corticosteroids [21], [22] and anti-inflammatory drugs [17], [23].

A new NAI, laninamivir octanoate, is a long-acting prodrug of laninamivir, and is currently available as Inavir® on the Japanese market [24], [25]. Laninamivir inhibits the neuraminidase activity of various influenza A and B viruses, including subtypes N1–N9, highly pathogenic avian influenza (H5N1) and A(H1N1)pdm09 viruses, and oseltamivir-resistant viruses [26], [27]. A single dose of laninamivir octanoate is sufficient to produce an anti-influenza effect, as shown by the long retention of laninamivir in mouse lungs [28]–[30]. In humans with influenza, the potential of a single inhalation of laninamivir octanoate has been demonstrated by clinical studies [31], [32].

Pulmonary surfactant produced by type II pneumocytes and Clara cells is constitutively secreted in the lumen of alveoli and alveolar ducts, and it contributes to inflation of the lung because of reduction of alveolar tension [33]. Pulmonary surfactant prevents collapse of the lung at the end of expiration by reducing the surface tension [34], [35]. Pulmonary surfactant consists of approximately 90% phospholipids and 10% surfactant-specific proteins designated SP-A, SP-B, SP-C and SP-D [36]. SP-A and SP-D play important roles in innate immunity via the promotion of phagocytosis by alveolar macrophages by binding to bacteria and viruses [37]. SP-B and SP-C reduce the surface tension of alveoli by their interfacial activity in association with the phospholipid content of pulmonary surfactant [38]. Surfactant proteins are not detectable in the serum of healthy individuals, but increase in association with interstitial pneumonia [39] and ARDS [40], [41] because of their leakage by abnormal permeability and/or destruction of the alveolar-capillary barrier, which is formed by type I pneumocytes of alveoli and endothelial cells of blood vessels.

We previously examined serial pathological changes of lungs in mice infected with a lethal dose of mouse-adapted influenza A/Puerto Rico/8/34 (PR8) virus (H1N1) strain [42]. All infected mice terminally died with DAD in the lung, which has been found in human autopsies of severe pneumonia and ARDS secondary to influenza virus infection. This previous study also suggested that pulmonary surfactant leaks from the alveolar lumen to capillaries in infected mice [42]. Administration of artificial surfactant or SP-D is known to provide a defense against influenza virus infection in vitro and in vivo via its anti-pathogenic activity [43], [44]. Therefore, in the present study, we examined whether the interfacial activity of artificial surfactant excluding SP-A and SP-D has a beneficial effect on mice infected with influenza virus under treatment with or without laninamivir octanoate.

## Results

### Decrease in pulmonary surfactant in infected lungs and administration of artificial surfactant to mice

**Figure 1:**
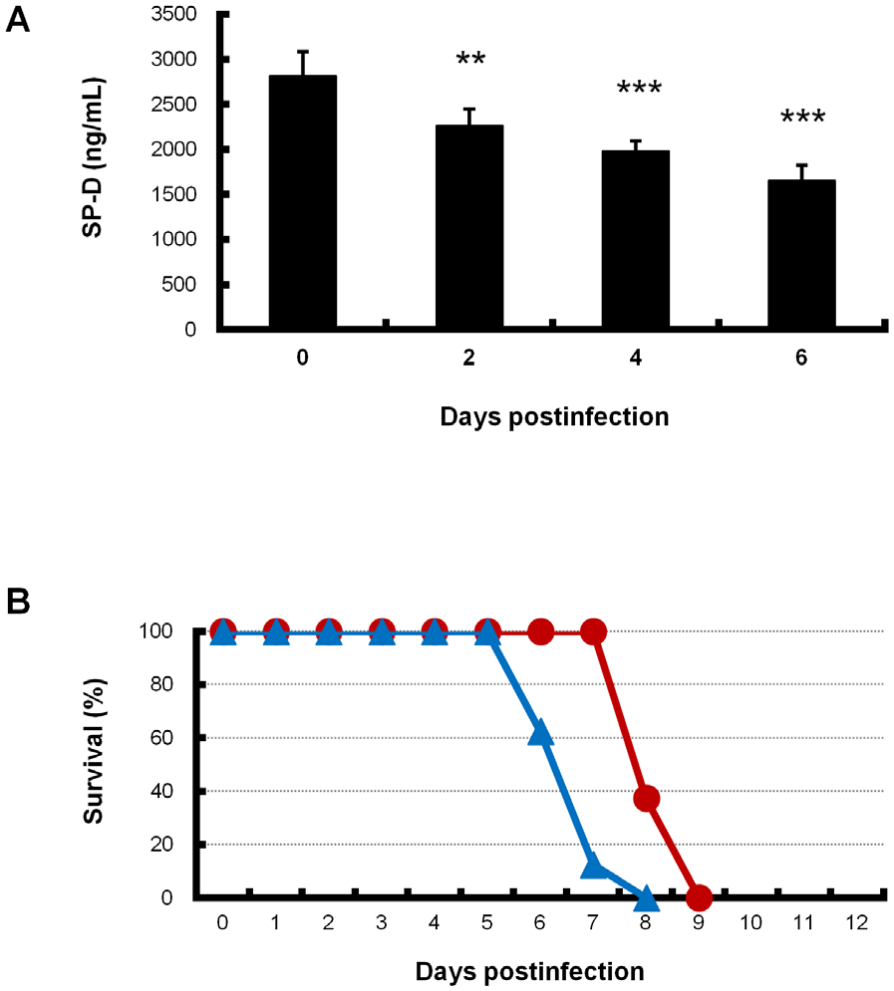
SP-D levels in infected lungs and survival of infected mice treated with artificial surfactant. (A) SP-D levels in lung homogenates from mice infected with the middle dose (5 MLD_50_) of PR8 virus were measured by ELISA. Infected mice were sacrificed at the indicated days, and then clarified lung homogenates (*n* = 5 or 6) were used. Differences in means ± SD and *p* values are shown. **p<0.01, ***p<0.001. (B) Survival curves of infected mice administered artificial surfactant are shown. Mice infected with a low dose (2 MLD_50_) of PR8 virus were additionally administered artificial surfactant (*n* = 8, red line) or normal saline solution (*n* = 8, blue line) intranasally once daily during 3–8 days postinfection. Significant differences in the survival rates of groups of mice treated with artificial surfactant or normal saline solution were analyzed by the log-rank original method. Percentage survival is shown.

Mice were intranasally inoculated with mouse-adapted PR8 virus under general anesthesia. Following infection, all mice had body weight recorded and were inspected daily for their general condition. In the first 2 days postinfection, no change in the general appearance of the infected mice was observed. Reduced activity, ruffled fur and difficulty breathing (tachypnea and labored respiration) were observed at 3 days postinfection. From 4 days postinfection, these conditions, including weight loss, diarrhea and cyanosis, gradually worsened. The infected mice were found dead after 5 days postinfection. Our previous study demonstrated a gradual increase in SP-A and SP-D, which are originally present in the alveolar lumen, in the serum of mice infected with a middle dose (5 50% mouse lethal dose [MLD_50_]) of PR8 virus in association with aggravation of viral pneumonia, suggesting leakage of pulmonary surfactant from the alveolar lumen to capillaries [42]. Therefore, in the present study, we quantitatively measured the level of SP-D in the lungs from mice infected with a middle dose (5 MLD_50_) of PR8 virus. SP-D in the lung homogenates gradually but significantly decreased from 2 to 6 days postinfection (Figure 1A). These results suggested a loss in absolute quantity of pulmonary surfactant. Therefore, exogenous artificial surfactant was administered to infected mice once daily from 3 days postinfection when alveolar collapse in the lung was observed [42]. Artificial surfactant administration provided no benefit with the middle dose of virus infection (data not shown). In contrast, when artificial surfactant was administered in low doses (1 and 2 MLD_50_) of viral infection, the mice survived 1–2 days longer than those administered with normal saline solution (*p* = 0.0005 and *p* = 0.0006, data not shown and Figure 1B, respectively). There was no significant difference in the general condition of mice with administration of artificial surfactant and normal saline solution.

### Combination therapy of artificial surfactant with laninamivir octanoate

**Figure 2:**
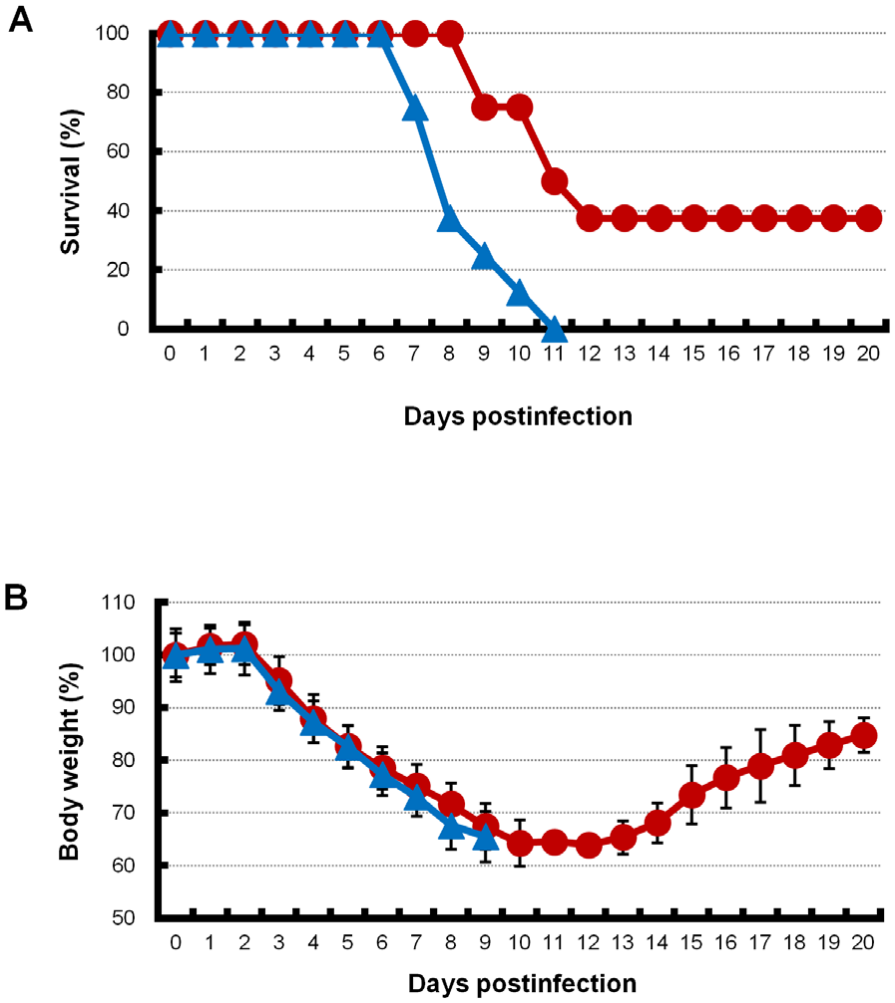
Survival curves and body weights of infected mice with combination therapy or monotherapy. (A) Survival curves of infected mice with combination therapy of artificial surfactant with laninamivir octanoate are shown. Mice infected with an extremely high dose (3741 MLD_50_) of PR8 virus were treated with laninamivir octanoate. The mice were additionally administered artificial surfactant (combination therapy, red line) or normal saline solution (monotherapy, blue line) intranasally once daily during 3–14 days postinfection. Significant differences in mouse survival rates between the combination therapy and monotherapy groups were analyzed by the log-rank original method. Experiments were independently repeated three times. Percentage survival in a representative experiment is shown. (B) Mouse body weight of the combination therapy (red line) and monotherapy (blue line) groups is shown. Experiments were independently repeated three times. The percentage of mouse body weight in a representative experiment is shown. Differences in means ± SD are shown.

To examine the effect of artificial surfactant administration under treatment with laninamivir octanoate, mice were intranasally inoculated with an extremely high dose (3741 MLD_50_) of PR8 virus. This viral dose was determined in our preliminary experiments because laninamivir octanoate rescued all infected mice in the low and middle doses of the virus. Mice with the extremely high dose of virus were administered laninamivir octanoate 2–3 h after virus inoculation. The mice were then divided into two groups. One group of mice was additionally administered artificial surfactant (combination therapy) once daily from 3 to 14 days postinfection. The other group was additionally administered normal saline solution (monotherapy). Thirty-eight percent of mice in the combination therapy group survived during the 20-day observation period (*p* = 0.003, Figure 2A). In contrast, all mice in the monotherapy group died within 11 days postinfection (Figure 2A). Incidentally, all infected mice treated with neither artificial surfactant nor laninamivir octanoate died within 7 days postinfection (data not shown). Body weights of mice in the combination therapy and monotherapy groups were similarly decreased (Figure 2B). The maximum improvement in survival rate was 50% in the combination therapy group compared with that in the monotherapy group when inoculated with a high dose (1247 MLD_50_) of PR8 virus (data not shown). To confirm the effect of the combination therapy of artificial surfactant with laninamivir octanoate, infected mice administered laninamivir octanoate were treated with 3- and 10-fold diluted artificial surfactant, and survival decreased with increasing dilution of artificial surfactant in a dose-dependent manner (data not shown). Moreover, the advantage of the combination therapy disappeared when artificial surfactant administration was started from 5 days postinfection (data not shown).

### Virus titers in the lungs, lung weight and immunological analysis

**Figure 3:**
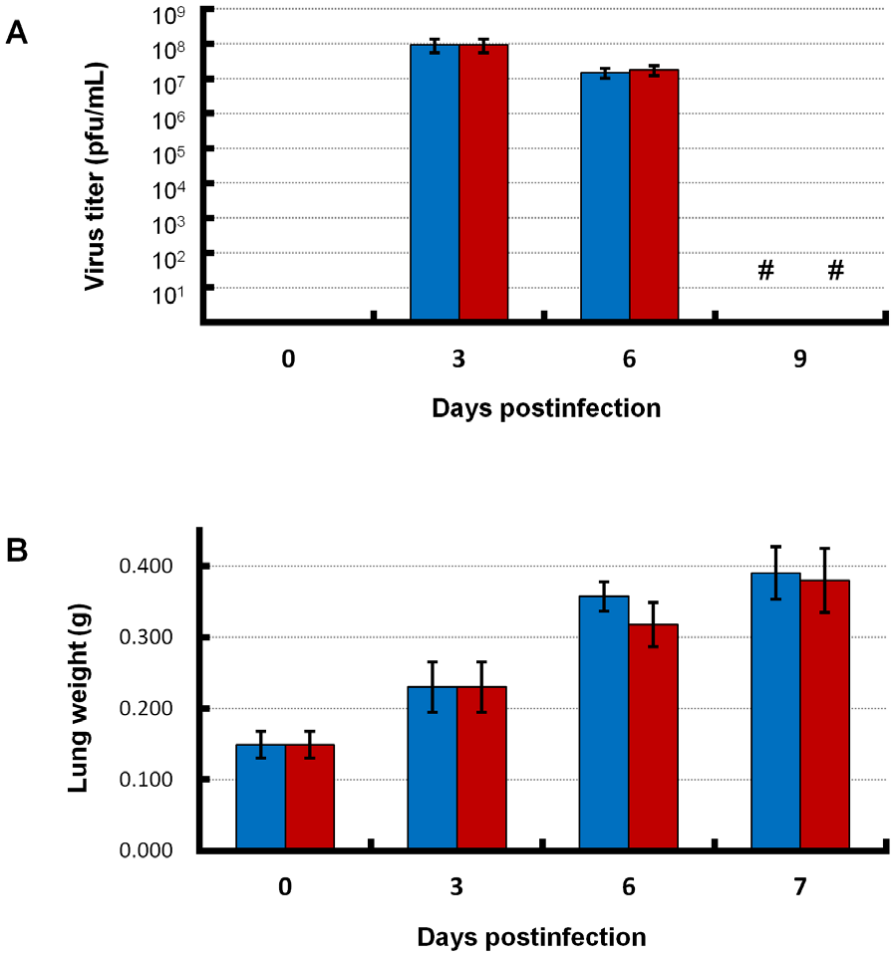
Virus titers in the lungs and lung weights of infected mice with combination therapy or monotherapy. (A) Virus titers in the lung homogenates of infected mice are shown. Mice infected with an extremely high dose (3741 MLD_50_) of PR8 virus were treated with laninamivir octanoate. The mice were additionally administered artificial surfactant (combination therapy, red bars) or normal saline solution (monotherapy, blue bars) intranasally once daily during 3–14 days postinfection. Four mice in each group were sacrificed at the indicated days postinfection and their lung homogenates were used for a plaque-forming assay. Mean ± SD viral titers are shown. The # symbol indicates under the detection limit. (B) Lung weights of four mice in the combination therapy (red bars) or monotherapy (blue bars) groups are shown. Mice were sacrificed at the indicated days postinfection and their lung weights were measured. Differences in means ± SD are shown.

**Figure 4:**
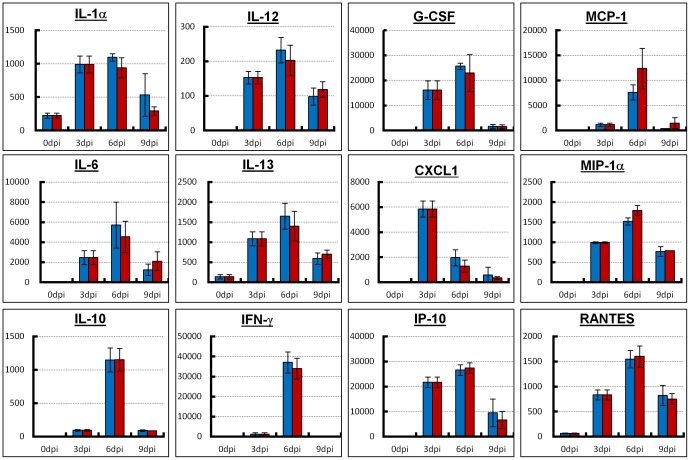
Cytokine and chemokine expression in lungs from infected mice with combination therapy or monotherapy. Mice intranasally inoculated with an extremely high dose (3741 MLD_50_) of PR8 virus were treated with laninamivir octanoate. The mice were additionally administered artificial surfactant (combination therapy, red bars) or normal saline solution (monotherapy, blue bars) intranasally once daily during 3–14 days postinfection. Three mice from each group were sacrificed at the indicated days postinfection and their lung homogenates were used to analyze the expression of 22 cytokines and chemokines using the MILLIPLEX MAP Panel. Only 12 cytokines and chemokines are shown; the remainder was under the limit of detection for the assay. The concentration in each panel is pg/ml. Differences in means ± SD are shown.

To determine the mechanism responsible for the improved survival in the combination therapy group, virus titers in mouse lungs were examined. There was no difference in the virus titer between the combination therapy and monotherapy groups (Figure 3A). To determine the severity of inflammation resulting from infection, lung weights of the infected mice were measured. Although lung weight gradually increased, there was no difference between the combination therapy and monotherapy groups (Figure 3B). Moreover, expression of inflammation-related cytokines and chemokines in the mouse lung between the combination therapy and monotherapy groups was measured using lung homogenates. Expression levels of interleukin (IL)-1α, IL-6, IL-10, IL-12, IL-13, interferon (IFN)-γ, granulocyte colony-stimulating factor (G-CSF), chemokine CXC ligand (CXCL)1, IFN-γ-induced protein (IP)-10, monocyte chemotactic protein (MCP)-1, macrophage inflammatory protein (MIP)-1α and regulated upon activation, normal T-cell expressed and secreted (RANTES) were similarly elevated between the combination therapy and monotherapy groups (Figure 4). However, IL-1β, IL-2, IL-4, IL-5, IL-7, IL-9, IL-15, IL-17, granulocyte-macrophage colony-stimulating factor (GM-CSF) and tumor necrosis factor (TNF)-α were not detected in either group.

### Pathological analysis of mouse lungs

**Figure 5:**
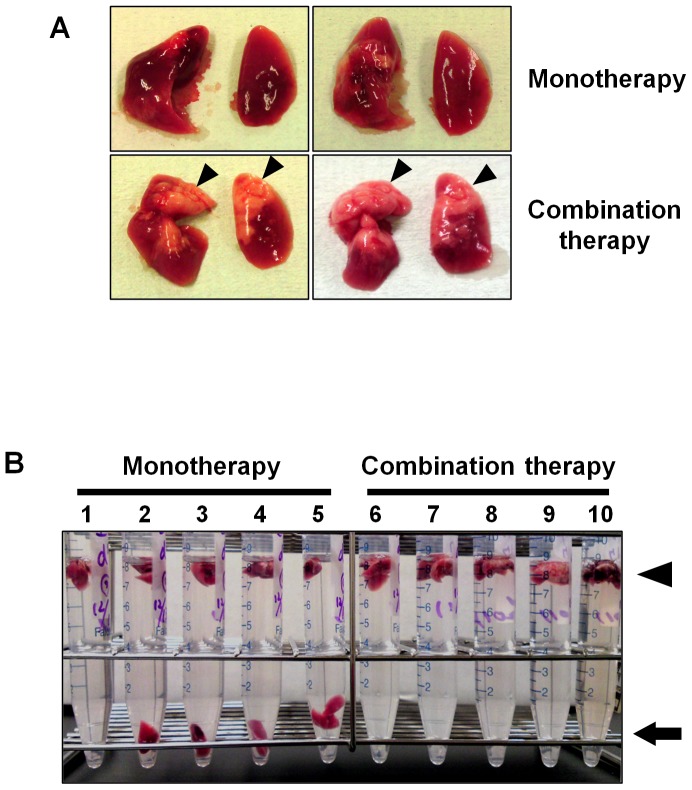
Gross pathology and hydrostatic test of lungs from infected mice with combination therapy or monotherapy. (A) Gross pathology of mouse lungs with combination therapy is shown. Mice intranasally inoculated with an extremely high dose (3741 MLD_50_) of PR8 virus were treated with laninamivir octanoate. The mice were additionally administered artificial surfactant (combination therapy, lower panels) or normal saline solution (monotherapy, upper panels) intranasally once daily during 3–14 days postinfection. Five mice in each group were sacrificed at 7 days postinfection, when the mouse survival rate in the monotherapy group was approximately 50%. Arrowheads indicate the area that appeared relatively healthy. (B) Hydrostatic lung test using mouse lungs from the monotherapy (tube number 1–5) and the combination therapy (tube number 6–10) groups is shown. The arrow indicates collapsing lungs (tube numbers 2–5). The arrowhead indicates floating lungs.

Postmortem pathological analysis showed that severe DAD with thick hyaline membranes was common in the lungs of the combination therapy and monotherapy groups, with no significant difference between them (data not shown). Mice in the combination therapy and monotherapy groups were sacrificed at 7 days postinfection, when the survival rate in the monotherapy group was approximately 50%. Macropathological examination showed that more lungs appeared relatively healthy in the combination therapy group than those in the monotherapy group (Figure 5A). Hydrostatic lung tests were performed to determine the degree of aeration, and all lungs excised from the combination therapy and monotherapy groups during 0–6 days postinfection floated (data not shown). At 7 days postinfection, in the monotherapy group, lungs from four of five mice sacrificed sank (Figure 5B). In contrast, no lungs in the combination therapy group sank (Figure 5B).

**Figure 6:**
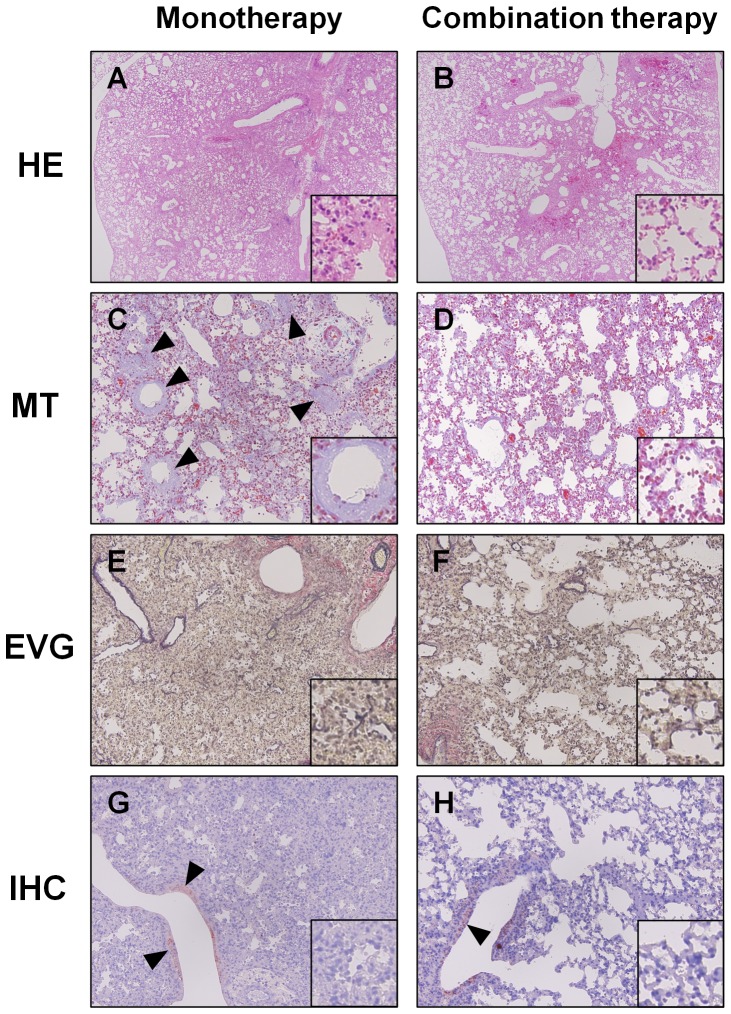
Histopathology of the lungs of infected mice with combination therapy or monotherapy. Mice intranasally inoculated with an extremely high dose (3741 MLD_50_) of PR8 virus were treated with laninamivir octanoate. The mice were additionally administered artificial surfactant (combination therapy, right panels) or normal saline solution (monotherapy, left panels) intranasally once daily during 3–14 days postinfection. Five mice in each group were sacrificed at 7 days postinfection, when mouse survival rate in the normal saline solution group was approximately 50%. Enlarged images are inserted in each panel. (A and B) Hematoxylin and eosin staining, magnification, 40×. (C) The hyaline membrane (arrowheads) was specifically stained pastel purple by Masson's Trichrome (MT) method in alveoli and alveolar ducts throughout the lungs in the control group. Magnification, 200×. (D) Little, if any, hyaline membrane was stained in mouse lungs from the surfactant group. Magnification, 200×. (E) Severe alveolar collapse can be seen. Elastica van Gieson (EVG) staining was used. Magnification, 200×. (F) Mild alveolar collapse can be seen. Magnification, 200×. (G and H) Immunohistochemistry (IHC) using anti-influenza virus polyclonal antibodies is shown. Red indicates influenza virus antigen. Antigen-positive cell debris (arrowheads) is located in the bronchioles. No antigen-positive cells or cell debris were found in pulmonary parenchyma. Magnification, 200×.

Micropathological examination showed that the air-filled area in the lungs was more extensive in the combination therapy group than in the monotherapy group (Figure 6A and B). Thick hyaline membranes were apparent in the lungs of the monotherapy group (Figure 6C). In contrast, a hyaline membrane was not observed in the lungs in the combination therapy group (Figure 6D). Alveolar collapse in the lungs of the combination therapy group was milder than that in the monotherapy group (Figure 6E and F). Immunohistochemistry revealed that there were no infected cells in the pulmonary parenchyma of either group of mice, and the same amount of infected cell debris was present within the lumen of the bronchioles in both groups (Figure 6G and H).

### Blood O_2_ pressure

**Figure 7:**
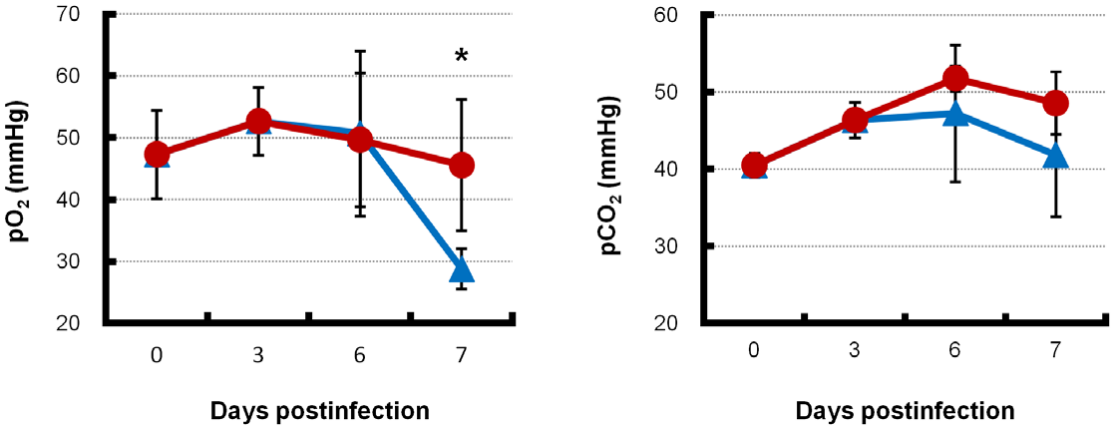
Blood O_2_ and CO_2_ pressures of infected mice with combination therapy or monotherapy. Mice intranasally inoculated with an extremely high dose (3741 MLD_50_) of PR8 virus were treated with laninamivir octanoate. The mice were additionally administered artificial surfactant (combination therapy, red lines) or normal saline solution (monotherapy, blue lines) intranasally once daily during 3–14 days postinfection. Mice (*n* = 3–6) in each group were sacrificed at the indicated days postinfection. Mice in each group were sacrificed at 7 days postinfection, when the mouse survival rate in the monotherapy group was approximately 50%. Blood was collected and immediately measured for O_2_ (left panel) and CO_2_ (right panel) pressures using the i-STAT portable blood gas analyzer. Differences in means ± SD and *p* values are shown. *p<0.05.

Blood gas pressure was determined to evaluate respiratory gas exchange in the lungs, which is a primary pulmonary function. The pH (data not shown) and CO_2_ pressure (Figure 7) in the blood were not significantly different between the combination therapy and monotherapy groups. Blood O_2_ pressure in the combination therapy and monotherapy groups was not different during 3 to 6 days postinfection. However, at 7 days postinfection, blood O_2_ pressure in the monotherapy group was significantly decreased compared with that in the combination therapy group (*p* = 0.021, Figure 7).

## Discussion

The present study demonstrated that combination therapy of laninamivir octanoate with artificial surfactant increased survival of mice suffering from influenza virus-induced severe pneumonia, while monotherapy of laninamivir octanoate did not improve survival. This increased survival was determined to be due to artificial surfactant inhibiting alveolar collapse and DAD formation in the lungs, thereby preserving lung function for oxygenation.

We previously showed a serially pathological process in the lungs that led to death in PR8-infected mice [42]. Interstitial pneumonia due to viral infection was found histopathologically within 2 days postinfection, and became severe during 2–6 days postinfection. In addition, DAD with hyaline membranes rapidly formed at 24–48 h before death. This rapid progression of DAD formation in the infected mice is consistent with the clinical time course for progression of existing diseases to ARDS in humans [8], [45]. It has also been found that the histopathological characteristics of DAD, hyaline membrane formation, inflammatory cell accumulation and pulmonary edema, in PR8-infected mouse lungs are identical to the characteristics of DAD in human cases of mortality involving severe viral pneumonia and ARDS induced by highly pathogenic avian influenza (H5N1) and A(H1N1)09 viruses [3]–[5], [7], [42]. Therefore, we consider that progression from interstitial pneumonia to DAD induced by influenza virus infection in the mouse resembles pulmonary changes in human cases. Moreover, our previous study demonstrated that interstitial pneumonia was observed in the lungs of live PR8-infected mice and DAD was only observed in dying and dead mice, suggesting that DAD formation was involved in the death of infected mice [42]. Based on these findings, we expected that inhibition of DAD formation by any medical intervention would improve survival of mice infected with influenza virus.

Neonatal respiratory distress syndrome (NRDS), also called infant respiratory distress syndrome and previously called hyaline membrane disease, is a syndrome in premature infants caused by developmental insufficiency of pulmonary surfactant production in the lungs [46]. NRDS affects approximately 1% of newborn infants and is the leading cause of death in preterm infants with alveolar collapse and hyaline membranes [47]. Current therapy of NRDS involves administration of exogenous artificial surfactant, which dramatically decreases mortality. In addition, a hyaline membrane is known to be composed of a mixture of cellular debris, immunoglobulin, fibrin and plasma proteins [48], [49]. This indicates an influx of plasma components, which are originally present in blood, to the alveolar lumen. Therefore, disruption of pulmonary surfactant is thought to be involved in the formation of alveolar collapse and hyaline membranes.

Our previous study demonstrated that the leakage of surfactant proteins to capillaries gradually increases in the infected mice in association with the aggravation of pneumonia up to 6 days postinfection [42]. This leakage continued to 20 days postinfection in the infected mice with treatment of oseltamivir (our unpublished data). Based on these results, we speculated that the amount of pulmonary surfactant in the alveolar lumen may decrease in infected lungs for at least 20 days after infection. A previous report found that when artificial surfactant was administered to mice infected with influenza virus only once at 3 days postinfection, there was an adverse effect on survival rate [50]. Therefore, in the present study, we administered artificial surfactant multiple times during the observation period. When multiple administration of artificial surfactant alone was investigated with a low viral dose inoculation, death in mice was delayed by a few days compared with that in normal saline controls (Figure 1). Although it is likely that multiple administration of artificial surfactant prevents a decrease in lung function by viral infection, damage of lung cells may continue because artificial surfactant does not inhibit viral proliferation, and then all infected mice eventually die. In the administration of artificial surfactant in the presence of laninamivir octanoate, the two agents acted synergistically, resulting in an improvement in survival of mice.

Our previous study found a delay of a few days between the peak of viral proliferation and death in mice [42]. In addition, in our present histopathological examination, the virus had already disappeared from the alveoli and alveolar ducts of living mice in the combination therapy and monotherapy groups at 7 days postinfection (Figure 6G and H). Furthermore, in the presence of an NAI, artificial surfactant increased survival rate in mice but did not diminish viral proliferation (Figures 2A and 3A). These observations suggest that viral replication itself is not directly correlated with death of infected mice. Moreover, as shown in our previous study, DAD formation is involved in the death of mice [42]. Therefore, it is hypothesized that progression from viral pneumonia to DAD formation is a multistep process. Infection and replication of influenza virus destroys type I/II pneumocytes in pulmonary parenchyma, leading to inflammation in the microenvironment, and this leads to destruction of the alveolar-capillary structure. Accumulation of infected cell debris in the lumen of alveoli, alveolar ducts and bronchioles, influx of protein-rich plasma components from capillaries into the alveolar lumen, and efflux of pulmonary surfactant from the alveolar lumen into the bloodstream then leads to quantitative and qualitative loss of pulmonary surfactant. The loss of pulmonary surfactant is associated with a decline in interfacial activity, resulting in low alveolar compliance, which accelerates formation of DAD with alveolar collapse and hyaline membrane. These pathological situations in pulmonary parenchyma finally disrupt gas exchange between the alveoli and capillaries, and ultimately lead to death. As mentioned above, at least in influenza virus infection, one of the factors responsible for increasing severity of viral pneumonia is thought to be the loss of pulmonary surfactant.

Treatment using exogenous SP-A and SP-D to influenza virus-infected mice has been previously attempted because of the efficacy of their anti-pathogenic functions in cooperation with alveolar macrophages [37], [51], [52]. However, in the present study, we did not consider this self-defense function by SP-A and SP-D. Artificial surfactant used in the present study consisted of lipoprotein, SP-B and SP-C only, because SP-A and SP-D were removed during the production process of artificial surfactant [53]. Therefore, artificial surfactant administration itself did not affect virus proliferation, inhibition of inflammation and a host immunological response. However, artificial surfactant administration inhibits formation of alveolar collapse and hyaline membranes, resulting in aeration of the lungs and constant blood O_2_ levels. Our results showed that interfacial activity of artificial surfactant improved alveolar gas exchange in severe pneumonia induced by influenza virus infection.

In humans, administration of artificial surfactant has been previously attempted for treatment of ARDS, but it was not effective [54]–[56]. However, a recent case report described that combination therapy with oseltamivir and artificial surfactant in a child infected with influenza virus produced favorable results [57]. Our previous study showed that alveolar collapses occurred at 2 days postinfection in mice [42]. In addition, the present study showed that commencement of artificial surfactant administration was crucial for treatment of severe pneumonia in the mouse infection model. Taken together, our studies and previous clinical reports suggest that for severe pneumonia in relation to influenza virus infection, the timing of artificial surfactant administration is important for successful treatment. Future studies are required to determine the timeline for successful administration of artificial surfactant to circumvent pathological decline. In humans, combination of NAIs and artificial surfactant could be a candidate for effective and preventive treatment of influenza virus-induced severe pneumonia, which does not improve with monotherapy of NAIs.

## Materials and Methods

### Mice and the virus

Six-week-old, specific pathogen-free female BALB/c mice (mean body weight ± SD, 20.53 g±0.91 g) were verified as being uncontaminated with pneumonia-causing pathogens, such as *Pasteurella pneumotropica*, *Mycoplasma pulmonis* and Sendai virus, (Japan SLC, Hamamatsu, Japan). Mouse-adapted PR8 virus, influenza A/Puerto Rico/8/34 (A/PR/8/34, H1N1), was provided by the National Institute of Infectious Diseases, Japan [58], and was grown once in the lungs of BALB/c mice. The lungs were excised and homogenized using a Multi-beads Shocker (Yasui Kikai, Osaka, Japan). The homogenate of the infected lungs was clarified by low speed centrifugation at 2500× *g* for 5 min at 4°C, and supernatant was used for virus inoculation. The PR8 virus titer was measured by a plaque-forming assay using Madin-Darby canine kidney (MDCK) cells.

### Cells, artificial surfactant, NAI and antibody

MDCK cells were obtained from the American Type Culture Collection (ATCC CCL-34). Cells were maintained in minimum essential medium (MEM) containing 10% fetal bovine serum, 50 U/ml penicillin, and 50 µg/ml streptomycin. Cells were cultured in 5% CO_2_ at 37°C. Artificial pulmonary surfactant was purchased from Tanabe-Mitsubishi (Osaka, Japan) and was suspended by normal saline solution in accordance with the manufacturer's instructions. The concentration of the suspended artificial surfactant solution was 30 mg/ml, which is equivalent to that used in humans. Laninamivir octanoate was synthesized by Daiichi Sankyo (Tokyo, Japan). The drug was diluted in normal saline solution in accordance with the manufacturer's instructions [31], [32]. The concentration of the diluted laninamivir octanoate was 267 µg/ml, which is equivalent to that used in humans. Rabbit anti-human influenza A, B virus polyclonal antibody (#M149; Takara, Tokyo, Japan) was used for immunohistochemical examination.

### Infection of mice with the virus

Mice were intranasally infected with the indicated doses of PR8 virus under general anesthesia with isoflurane. Following infection, all mice had body weight measured and were inspected daily to evaluate their general condition during the 20-day observation period. The condition of the mice gradually became worse, but it was not severe enough to euthanize the mice in accordance with our guidelines. The infected mice were found dead after 5 days postinfection. In experiments with artificial surfactant administration in the absence of laninamivir octanoate, mice (*n* = 16) were intranasally infected with 50 µl viral solution containing a low dose (2 MLD_50_) of PR8 virus under anesthesia and divided into two groups (*n* = 8 per group). In the combination therapy experiments using laninamivir octanoate and artificial surfactant, mice (*n* = 16) inoculated with 50 µl viral solution containing an extremely high dose (3741 MLD_50_) of PR8 virus were intranasally administered 50 µl laninamivir octanoate 2–3 h after virus inoculation under anesthesia and divided into two groups (*n* = 8 per group). Mice were additionally administered 45 µl artificial surfactant or normal saline solution intranasally once daily at 3–14 days postinfection under anesthesia. Following infection, all mice had body weight measured and were inspected daily to examine their general condition. In experiments to determine viral titer, lung weight, cytokine/chemokine levels, as well as pathological analysis and blood gas analysis, an approximately 2–3 times higher number of mice was used compared with the number of mice used for the survival experiment to sample a sufficient number of mice during the 6–9 days of postinfection. Mice were selected at random and were sacrificed at the indicated days in these experiments. For the selection of mice, their symptoms were not taken into account to prevent selection bias. In the experiments for determining SP-D levels in lung tissues, mice (*n* = 21) inoculated with 50 µl viral solution containing a middle dose (5 MLD_50_) of PR8 virus were sacrificed at 0, 2, 4 and 6 days postinfection, and lung homogenates were used for SP-D ELISA.

### SP-D ELISA

To determine the amount of SP-D in mouse lungs infected with PR8 virus, an SP-D ELISA was performed according to the manufacturer's instructions (Yamasa, Choshi, Japan) [59]. Infected mice were sacrificed at 0, 2, 4 and 6 days postinfection and lungs were excised and homogenized using a Multi-beads Shocker. Lung homogenates were clarified by low speed centrifugation at 2500× *g* for 5 min at 4°C and then middle speed centrifugation at 13000× *g* for 5 min at 4°C. Supernatant was purified using the Aurum serum protein mini kit (Bio-Rad, Hercules, CA) to remove albumin and immunoglobulin, and was used for ELISA.

### Plaque-forming assay

To measure virus titers in the lungs of infected mice, a plaque-forming assay was performed, as previously described [25]. In brief, infected mice were sacrificed at 0, 3, 6 and 9 days postinfection. Excised lungs were homogenized using a Multi-beads Shocker and clarified by centrifugation at 2500× *g* for 5 min at 4°C. The clarified supernatants containing the virus were serially diluted in MEM containing 0.2% bovine serum albumin, 2 mM L-glutamine, 50 U/ml penicillin, and 50 µg/ml streptomycin. Dilutions of the virus were used to infect MDCK cell monolayers for 1 h at 37°C. Cells were washed with phosphate-buffered saline (PBS) once to remove free viruses, overlaid with modified MEM containing 0.6% agar, 0.2% bovine serum albumin, 0.01% DEAE–dextran, 25 mM HEPES, and 1 µg/ml trypsin, and incubated at 37°C. After incubation for 2 days, the monolayer cells were stained with crystal violet solution (0.095% crystal violet and 19% methanol).

### Immunological analysis of the lungs

Mouse lung homogenates were clarified by low-speed (2500× *g*, 5 min, 4°C) and middle speed (13000× *g*, 5 min, 4°C) centrifugation. Supernatants were used for immunological analysis using the MILLIPLEX MAP Panel (Millipore, Billerica, MA) for 22 mouse cytokines and chemokines (IL-1α, IL-1β, IL-2, IL-4, IL-5, IL-6, IL-7, IL-9, IL-10, IL-12, IL-13, IL-15, IL-17, G-CSF, GM-CSF, IFN-γ, IP-10, CXCL1, MCP-1, MIP-1α, RANTES and TNF-α) and analyzed using Luminex 100 (Millipore).

### Histopathological analysis

Mouse lungs were fixed in 4% paraformaldehyde, embedded in paraffin, sectioned and stained with hematoxylin and eosin. Masson's Trichrome staining [60] was performed to visualize hyaline membrane formation in tissue sections. The Elastica van Gieson method [61] was performed to visualize crumpling alveoli without air spaces, because elastic fibers in alveolar septa were stained black. Immunohistochemical analysis was performed to detect influenza virus antigens. In brief, histopathological slides were deparaffinized and treated with methanol containing 3% hydrogen peroxide to block endogenous peroxidase. Slides were incubated with rabbit anti-human influenza A, B virus polyclonal antibody [62] and were treated with HistoMouse Max (Invitrogen, San Diego, CA), according to the manufacturer's instructions [63]. Slides were viewed using an Olympus BX51 microscope and software DP controller to capture images (Olympus, Tokyo, Japan).

### Blood gas analysis

Mice were decapitated to obtain blood samples for analysis of blood gas levels. The blood was collected using capillary glass tubes containing heparin, and immediately applied to a G3+ cartridge. Measurement of the levels of blood O_2_ and CO_2_ pressures and pH was performed using an i-STAT portable blood gas analyzer (Abbott, Princeton, NJ), as described previously [64].

### Statistical analysis

All data are expressed as means ± SD. Differences between groups were assessed using the Student's *t*-test, and *p*<0.05 was considered statistically significant. Survival rates were analyzed by the Kaplan-Meier method and its significance was evaluated by the original log-rank method.

### Ethics

Our research protocol with mice and mouse husbandry conditions followed the Regulations for Animal Care and Use of Daiichi Sankyo Co., Ltd. The protocol was approved by the Institutional Animal Care and Use Committee of Daiichi Sankyo Co., Ltd. (approval ID: C 0083), and was waived by the National Center for Global Health and Medicine.
